# The comparative diagnostic accuracy of the Mini Mental State Examination (MMSE) and the General Practitioner assessment of Cognition (GPCOG) for identifying dementia in primary care: a systematic review protocol

**DOI:** 10.1186/s41512-017-0014-1

**Published:** 2017-06-02

**Authors:** Harriet A. Hunt, Sanne Van Kampen, Yemisi Takwoingi, David J. Llewellyn, Mark Pearson, Christopher J. Hyde

**Affiliations:** 1 National Institute for Health Research (NIHR) Collaboration for Leadership in Applied Health Research and Care South West Peninsula, Institute of Health Research, University of Exeter Medical School, St Luke’s Campus, Exeter, Devon EX1 1TE UK; 2 National Institute for Health Research (NIHR) Collaboration for Leadership in Applied Health Research and Care South West Peninsula, Plymouth University Peninsula Schools of Medicine and Dentistry, ITTC building, Tamar Science Park, Drake Circus, Plymouth, Devon PL4 8AA UK; 3 Institute of Applied Health Research, College of Medical and Dental Sciences, University of Birmingham, Edgbaston, Birmingham, B15 2TT UK; 4 University of Exeter Medical School, St Luke’s Campus, Exeter, Devon EX1 1TE UK; 5 Institute of Health Research, University of Exeter Medical School, St Luke’s Campus, Exeter, Devon EX1 1TE UK

**Keywords:** Dementia, Brief cognitive assessment, Diagnostic accuracy, Primary care, Systematic review

## Abstract

**Background:**

Improved dementia identification is a global health priority, and general practitioners (GPs) are often the first point of contact for people with concerns about their cognition. However, GPs often express uncertainty in using assessment tools and the evidence based on which tests are most accurate in identifying dementia is unclear. In particular, there is little certainty around how the accuracy of available brief cognitive assessments compares within a clinical family practice setting.

Grounded in existing brief cognitive assessment evidence, we will compare the diagnostic test accuracy of the Mini Mental State Examination (MMSE) to the General Practitioner Assessment of Cognition (GPCOG) against the best available reference standard when used within a family practice setting.

**Methods:**

We will employ robust systematic review methods to assess studies of diagnostic accuracy where both the MMSE and GPCOG have been evaluated as direct comparisons, i.e. within the same study population. This approach will enable us to minimise between-study heterogeneity, to eliminate the risk of bias due to confounding and increase the opportunity to make clinically useful and useable comparisons of diagnostic accuracy across both the MMSE and GPCOG. This systematic review will be conducted using a pragmatic search strategy, refining searches that build upon studies identified as part of our overview of systematic reviews of the diagnostic accuracy of brief cognitive assessments for identifying dementia in primary care.

**Discussion:**

Through this systematic review, we aim to improve existing evidence on how the diagnostic accuracy of MMSE and GPCOG compares when used to identify dementia within the family practice setting. We also aim to make clinical practice recommendations based upon the variations in diagnostic accuracy identified between the MMSE and GPCOG.

**Electronic supplementary material:**

The online version of this article (doi:10.1186/s41512-017-0014-1) contains supplementary material, which is available to authorized users.

## Background

Improved dementia diagnosis is a global health priority of international bodies such as the World Health Organization [1] and the G8 [2]. Existing delays in the diagnostic pathway have led to debates around case finding and targeted screening within primary care [3–6]. General practitioners (GPs) are often the first point of contact for people with concerns about their cognition, yet GPs often express uncertainty in using assessment tools alongside concerns around the consequences of misdiagnosing dementia [5, 7, 8]. In established healthcare systems, guidelines on the most accurate brief cognitive assessment for identifying dementia in primary care are inconsistent and variable in their specific recommendations. Whilst there is variation in guidance on thresholds, accuracy and suitability of test within different populations, guidelines often feature the same subset of brief cognitive assessments. Examples are available in the UK from the National Institute for Health and Care Excellence [9] and the Royal College of Psychiatrists [10], and in the Netherlands from the Huisartsen Genootschap (GP Society) [11]. These all include the Mini Mental State Examination (MMSE) and the General Practitioner Assessment of Cognition (GPCOG).

A number of systematic reviews [12–18] have explored the individual diagnostic accuracy of brief cognitive assessments for dementia in isolation, and across a range of populations and settings. In an overview of systematic reviews of the diagnostic accuracy of brief cognitive assessments for identifying dementia in primary care, we identified two brief cognitive assessments that can be compared to identify the test with better diagnostic performance. These tests were two of the three most frequently assessed brief cognitive assessments within the 13 included systematic reviews [12, 13, 19–29] with the MMSE featuring in 8 reviews and the GPCOG featuring in 4 reviews. The clock drawing test (CDT) was the third most frequently assessed tool, featuring in 4 reviews. We judged this to be less comparable to the MMSE in terms of administration complexity, timing and domains assessed, compared to the GPCOG. As the most frequently assessed test within our overview, the MMSE is also included as one of the index tests within this review as, whilst copyright restrictions are now enforced, it remains one of the most popular brief cognitive assessments employed in practice [27, 30]. The MMSE is based on a 30-point scale of 11 questions testing five domains of cognitive function (orientation, registration, attention and calculation, recall and language) [31]. The GPCOG was the second most frequently assessed index test within our overview. The GPCOG is a publicly available test that has two sections: a patient examination (GPCOG-patient) with a maximum score of 9 (optimum performance) covering time orientation, clock drawing, reporting recent events and a word-recall task, and an optional informant questionnaire (GPCOG-informant) with a maximum score of 6 with questions assessing the patient’s memory of recent events and their executive function [32]. In comparison, the CDT is a standard assessment where the patient is asked to draw a clock face marking the hours and then draw the hour and minute hands to correctly indicate a specific time (e.g. quarter past 3). There are a number of scoring approaches, but the Shulman method uses a 6-point scoring system [33] whilst the Sunderland method uses a 10-point system [34]. Taking into consideration the ubiquity of the index tests, the comparability of the tests mentioned above and their common use within guidelines, we have chosen to compare the MMSE against the GPCOG as index tests within this systematic review. Therefore, the aim of this systematic review is to compare the diagnostic accuracy evidence of the MMSE and the GPCOG for identifying dementia, particularly within a primary care setting and using direct (within study) comparisons.

This use of direct comparisons should reduce between-study heterogeneity and allow us to draw firm conclusions about the comparative accuracy of these brief cognitive assessments within the same or similar populations [35, 36]. To our knowledge, this type of systematic review has not previously been conducted to compare the accuracy of brief cognitive assessments for identifying dementia.

This evidence will contribute strongly to clinical practice and policy making by demonstrating the presence or absence of superiority in the diagnostic accuracy of GPCOG relative to that of MMSE for identifying dementia in primary care.

## Methods

The primary outcome is the comparative accuracy of the two tests assessed via direct comparisons, i.e. the diagnostic accuracy of the two tests are compared within the same population in a study (comparative study).

The secondary outcome of the review is to identify other common test-related factors identified by included studies, such as ease of administration or administration time. Whilst beyond our primary focus of test accuracy, these other factors may contribute to the overall usefulness of the tests when applied in a primary care setting, and we will incorporate them in our findings in order to make useful research and clinical recommendations.

This systematic review will be conducted using a pragmatic search strategy, refining searches that build upon studies identified as part of our overview of systematic reviews of the diagnostic accuracy of brief cognitive assessments for identifying dementia in primary care. Further details are given below (PROSPERO reference 42015022078).

### Overview search methods

To build the search database for the overview of systematic reviews of the diagnostic accuracy of brief cognitive assessments for identifying dementia in primary care, we searched the Cochrane Database of Systematic Reviews, EMBASE, MEDLINE and PsychINFO for systematic reviews from inception until August 2015. Search strategies are shown in the Additional file 1. According to best searching practice for diagnostic accuracy reviews, we applied no date or language restrictions, and where reviews were updated, we used the latest version available. Additional papers were identified through Zetoc alerts and incorporated at the title and abstract screening phase. We ran updated searches on the Cochrane Database of Systematic Reviews in February 2016.

#### Eligibility criteria

Adults aged 18 years or over recruited from a primary care or general practice population were included, and we did not exclude patients who were selected on the basis of an existing diagnosis or condition which might reasonably be expected to feature in primary care (e.g. stroke).

The target condition was all-cause (non-differentiated) dementia. We also included reviews that focused specifically on differentiated forms of dementia such as Alzheimer’s disease, vascular dementia and dementia with Lewy bodies. We excluded reviews that focused on mild cognitive impairment (MCI). Where reviews investigated both dementia and MCI, we extracted data referring to dementia and excluded data referring solely to MCI.

### Identification of studies for this systematic review

To identify eligible studies for this systematic review, we will first assess the 13 systematic reviews included within our overview review (methods described above) and identify included reviews that contained studies including direct comparisons of the diagnostic accuracy of MMSE and GPCOG for identifying dementia in primary care. Once we have identified these studies, we will carry out citation tracking via Google Scholar, i.e. clicking on the appropriate link (e.g. “cited by 15”) to view details on the articles that have cited the original study. We will also use these initial studies to conduct snowball searching, i.e. checking the bibliographies for relevant original studies for possible inclusion within this systematic review. We will use Zetoc alerts to proactively identify recent studies published that meet our criteria (using the terms “MMSE”, “GPCOG”, “test accuracy” and “dementia”). Finally, when we have identified studies using the above methods, we will conduct a traditional search taking a start date 1 year prior to the most recently published identified study up to the current day, using MEDLINE, EMBASE and PsychINFO databases. The rationale is that this search will cover the maximum period of time not covered in the overview review searches with some date overlap to ensure all potential sources are included, using the most efficient means to identify the most recent evidence. This will also enable us to confirm whether we identified all relevant studies via the overview searches.

### Index tests

The index tests are the MMSE [31] and the GPCOG [32]. The MMSE is one of the most widely used brief cognitive assessments currently used, and development of the GPCOG has been independent to the development of the MMSE.

The conventional threshold for the MMSE is 24 (also shown as <24), where out of a maximum possible 30 points, scores below 24 indicate impairment [22]. The GPCOG comprises of two sections: the section completed by the individual being assessed, known as GPCOG-patient, and an optional section for a relative or friend to complete (if present) known as GPCOG-informant. GPCOG-patient has 9 items with possible total scores of between 0 (indicating severe impairment) and 9 (indicating no impairment). GPCOG-informant has 6 items with possible total scores of between 0 (indicating severe impairment) and 6 (indicating no impairment). GPCOG-patient can be conducted by itself, with a conventional threshold of 8 out of 9 (<8). If informants are available, a score of GPCOG-patient between 5 and 8 precipitates the GPCOG-informant and the scores are combined (“GPCOG-total”) with a conventional threshold of 11 out of a maximum 15 (<11). If no informant is available, the conventional threshold of 8 stands. It is also possible to conduct a staged GPCOG assessment where GPCOG-informant is only required if GPCOG-patient is scored between 5 and 8 out of 9. This is known as “GPCOG Two stage”.

For our assessment, we will stratify GPCOG into 3 types of test: GPCOG-patient with a threshold of <8, GPCOG-total with a threshold of <11 and GPCOG Two stage [37].

### Reference standard

There is currently no gold standard test for identifying dementia in primary care. We will accept reference standards consisting of the following tools alone, clinical diagnosis alone or clinical diagnosis combined with one or a combination of the following assessment tools:

- Diagnostic and Statistical Manual (DSM) III/III-R/IV/IV-R,
- Clinical Dementia Rating (CDR),
- International Classification of Diseases (ICD) 10,
- Geriatric Mental State–Automated Geriatric Examination for Computer Assisted Taxonomy (GMS-AGECAT),
- Cambridge Mental Disorders of the Elderly Examination (CAMDEX),
- International Psychogeriatric Association World Health Organization (IPA-WHO) criteria.

Reference standards are selected on the basis of many variables such as common practice within individual clinics, practitioner preference, specialisation and experience of healthcare professionals and practice managers and are subject to changes in cost and fashion. Many of the globally accepted reference standards such as the World Health Organization-supported ICD and the DSM produced by the American Psychiatric Association are updated regularly; the DSM-5 (sometimes referred to as DSM-V) was released in 2013 [38], and the ICD-11 is due for release by 2018 [39].

### Data extraction, selection and coding

All sources will be managed using the latest version of EndNote software. Two reviewers will pilot the screening for titles and abstracts on the first 15 sources, and we will write screening notes to help with title/abstract and full-text screening. Title/abstract and full-text screening will be conducted by the same two reviewers, and a third reviewer will resolve any disagreements.

A bespoke data abstraction form will be piloted by two reviewers using two included studies. Key data extracted will include characteristics of included systematic reviews (references and author details, overall goal of review, date review conducted, date published, participant details), included study details (such as authors, year of study, date of publication, country of study, outcomes reported, test timings) and general review limitations as well as components of the 2 × 2 table (TP, FP, TN, FN) or other accuracy data such as sensitivity, specificity and disease prevalence if raw numbers are not available. The data abstraction form will be accompanied by a briefing document explaining how it should be used. Data will be abstracted by one reviewer, spot-checked by a second, with a third reviewer acting as moderator if necessary.

### Assessment of methodological quality

We will use the QUADAS-2 [40] tool to assess methodological quality of diagnostic accuracy studies for systematic reviews. Whilst this tool is developed for studies focussing on a single index test, we will assess the suitability of using the tool for studies that focus on direct comparisons of two index tests by piloting the QUADAS-2 tool on one of the included studies. We will tailor QUADAS-2 in line with suitability in assessing quality of studies using direct comparisons, for example assessing the reference standard against MMSE and then the reference standard against GPCOG.

### Data synthesis and analysis

Study of specific estimates of the sensitivity and specificity (and their 95% confidence intervals) of GPCOG and MMSE will be presented graphically on a forest plot. We will also use these forest plots and summary receiver operating characteristic (SROC) plots to visually explore heterogeneity.

We will consider possible sub-group analyses investigating, for example tests using lower and higher thresholds. Other aspects that may be suitable for investigating through sub-grouping could include variations in population details such as prevalence, and variations in cases and control groups (e.g. confirmed dementia, probably dementia, people with memory problems, healthy people).

We will perform meta-analysis if the quantity and nature of the included studies permit. Again, if data allow, we will use a hierarchical meta-regression model with test type as a covariate to estimate and compare SROC curves or summary points [36]. A priori uncertainty about thresholds for determining test positivity and the likelihood of implicit thresholds suggests estimation of SROC curves using a hierarchical SROC (HSROC) meta-regression model may be preferable [41]. However, we will consider using a bivariate meta-regression model to estimate and compare summary points [42, 43] if studies use a common threshold.

We will create a summary of result table with additional summary tables of subgroup results (potential subgroups listed above) if relevant. If feasible and appropriate, we will consider translating any summary results into natural frequencies and other metrics such as predictive values to help improve understanding by readers.

We will not assess reporting bias because its impact on diagnostic accuracy is unclear, and the tools for investigating it are in the early stages of development [44].

## Discussion

We do not foresee any practical or operational issues with the conduct of this systematic review. All differences between the protocol and systematic review will be reported in the full systematic review.

## Additional file

Additional file 1: Search strategy, formatted for EMBASE (OVID). (DOCX 24 kb)

## Abbreviations

CAMDEX: Cambridge Mental Disorders of the Elderly Examination
CDR: Clinical Dementia Rating
CDT: Clock drawing test
DSM: Diagnostic and Statistical Manual
EMBASE: Excerpta Medica dataBASE
FN: False negative
FP: False positive
G8: Group of Eight (consensus policy group of highly industrialised nations: France, Germany, UK, Japan, USA, Canada, Russia)
GMS-AGECAT: Geriatric Mental State–Automated Geriatric Examination for Computer Assisted Taxonomy
GP: General practitioner
GPCOG: General Practitioner Assessment of Cognition
HSROC: Hierarchical summary receiver operating characteristic
ICD: International Classification of Diseases
IPA-WHO: International Psychogeriatric Association World Health Organization
MCI: Mild cognitive impairment
MEDLINE: Medical Literature Analysis and Retrieval System Online
MMSE: Mini Mental State Examination
NHS: National Health Service
NIHR: National Institute for Health Research
PROSPERO: International prospective register of systematic reviews
PsychINFO: Bibliographic database of psychological research
QUADAS/QUADAS-2: Quality Assessment for Diagnostic Assessment Studies/2nd version
SROC: Summary receiver operating characteristic
TN: True negative
TP: True positive
WHO: World Health Organization
