# Supplementary material for: The comparative diagnostic accuracy of the Mini Mental State Examination (MMSE) and the General Practitioner assessment of Cognition (GPCOG) for identifying dementia in primary care: a systematic review protocol
Source: Diagn Progn Res. 2017 Jun 2;1:14. doi: 10.1186/s41512-017-0014-1 (PMC6460787; doi:10.1186/s41512-017-0014-1)
Supplement: Supplementary file 1 — Search strategy, formatted for EMBASE (OVID). (DOCX 24 kb) [file 41512_2017_14_MOESM1_ESM.docx]

## Supplementary files

### Search strategy, formatted for EMBASE (OVID)

Embase 1974 to 2015 August 21

| 1 | dementia/ or aids dementia complex/ or alzheimer disease/ or aphasia, primary progressive/ or creutzfeldt-jakob syndrome/ or dementia, vascular/ or diffuse neurofibrillary tangles with calcification/ or frontotemporal lobar degeneration/ or huntington disease/ or kluver-bucy syndrome/ or lewy body disease/ | 225474 |
| --- | --- | --- |
| 2 | exp dementia/ | 252956 |
| 3 | exp dementia assessment/ | 23039 |
| 4 | exp clinical dementia rating/ | 1011 |
| 5 | exp memory disorder/ | 56295 |
| 6 | dementia.ti,ab. | 101033 |
| 7 | alzheimer*.ti,ab. | 131501 |
| 8 | 1 or 2 or 3 or 4 or 6 or 7 | 292776 |
| 9 | systematic*.ti,ab. | 317342 |
| 10 | meta-analysis.ti,ab. | 87144 |
| 11 | "systematic review".ti,ab. | 76490 |
| 12 | exp review/ | 2082052 |
| 13 | (literature adj3 review$).ti,ab. | 238313 |
| 14 | exp meta analysis/ | 97571 |
| 15 | exp "Systematic Review"/ | 93826 |
| 16 | or/12-15 | 2316420 |
| 17 | (medline or medlars or embase or pubmed or cinahl or amed or psychlit or psyclit or psychinfo or psycinfo or scisearch or cochrane).ti,ab. | 133763 |
| 18 | RETRACTED ARTICLE/ | 7620 |
| 19 | 17 or 18 | 141333 |
| 20 | 16 and 19 | 103832 |
| 21 | (systematic$ adj2 (review$ or overview)).ti,ab. | 95715 |
| 22 | (meta?anal$ or meta anal$ or meta-anal$ or metaanal$ or metanal$).ti,ab. | 105476 |
| 23 | 20 or 21 or 22 | 211820 |
| 24 | diagnosis/ | 1066933 |
| 25 | "sensitivity and specificity"/ | 231631 |
| 26 | 9 or 10 or 11 or 24 or 25 | 1642613 |
| 27 | brief cognitive tests.ti,ab. | 66 |
| 28 | cognitive screen*.ti,ab. | 1402 |
| 29 | ("screening test*" adj2 (dement* or alzheimer*)).ti,ab. | 222 |
| 30 | cog*.ti,ab. | 368545 |
| 31 | 27 or 28 or 29 or 30 | 368615 |
| 32 | "primary care".ti,ab. | 100669 |
| 33 | "general practic*".ti,ab. | 40969 |
| 34 | "GP".ti,ab. | 41972 |
| 35 | 32 or 33 or 34 | 169068 |
| 36 | 8 and 26 and 31 | 9009 |

N.B. systematic review search filter

- exp review/
- (literature adj3 review$).ti,ab.
- exp meta analysis/
- exp "Systematic Review"/
- or/1-4
- (medline or medlars or embase or pubmed or cinahl or amed or psychlit or psyclit or psychinfo or psycinfo or scisearch or cochrane).ti,ab.
- RETRACTED ARTICLE/
- 6 or 7
- 5 and 8
- (systematic$ adj2 (review$ or overview)).ti,ab.
- (meta?anal$ or meta anal$ or meta-anal$ or metaanal$ or metanal$).ti,ab.
- 9 or 10 or 11

From [BMJ Clinical Evidence strategy](http://clinicalevidence.bmj.com/x/set/static/ebm/learn/665076.html) [undated] [Ovid] was used.

### Search strategy, formatted for OVID MEDLINE

Ovid MEDLINE(R) 1946 to August Week 2 2015

| 1 | dementia/ or aids dementia complex/ or alzheimer disease/ or aphasia, primary progressive/ or creutzfeldt-jakob syndrome/ or dementia, vascular/ or diffuse neurofibrillary tangles with calcification/ or frontotemporal lobar degeneration/ or huntington disease/ or kluver-bucy syndrome/ or lewy body disease/ | 127632 |
| --- | --- | --- |
| 2 | Dementia/ | 39509 |
| 3 | exp dementia/ | 129660 |
| 4 | exp memory disorder/ | 24008 |
| 5 | dementia.ti,ab. | 67035 |
| 6 | alzheimer*.ti,ab. | 92544 |
| 7 | 1 or 2 or 3 or 4 or 5 or 6 | 188618 |
| 8 | systematic*.ti,ab. | 221142 |
| 9 | meta-analysis.ti,ab. | 57552 |
| 10 | "systematic review".ti,ab. | 50417 |
| 11 | (((comprehensive* or integrative or systematic*) adj3 (bibliographic* or review* or literature)) or (meta-analy* or metaanaly* or "research synthesis" or ((information or data) adj3 synthesis) or (data adj2 extract*))).ti,ab. or (cinahl or (cochrane adj3 trial*) or embase or medline or psyclit or (psycinfo not "psycinfo database") or pubmed or scopus or "sociological abstracts" or "web of science").ab. or ("cochrane database of systematic reviews" or evidence report technology assessment or evidence report technology assessment summary).jn. or Evidence Report: Technology Assessment*.jn. or ((review adj5 (rationale or evidence)).ti,ab. and review.pt.) or meta-analysis as topic/ or Meta-Analysis.pt. | 212532 |
| 12 | diagnosis/ | 17054 |
| 13 | "sensitivity and specificity"/ | 295160 |
| 14 | 8 or 9 or 10 or 12 or 13 | 562806 |
| 15 | brief cognitive tests.ti,ab. | 41 |
| 16 | cognitive screen*.ti,ab. | 735 |
| 17 | ("screening test*" adj2 (dement* or alzheimer*)).ti,ab. | 161 |
| 18 | cog*.ti,ab. | 243367 |
| 19 | 15 or 16 or 17 or 18 | 243415 |
| 20 | "primary care".ti,ab. | 71023 |
| 21 | "general practic*".ti,ab. | 32013 |
| 22 | "GP".ti,ab. | 27969 |
| 23 | 20 or 21 or 22 | 121226 |
| 24 | 7 and 14 and 19 | 2660 |

N.B. systematic review search filter

(((comprehensive* or integrative or systematic*) adj3 (bibliographic* or review* or literature)) or (meta-analy* or metaanaly* or "research synthesis" or ((information or data) adj3 synthesis) or (data adj2 extract*))).ti,ab. or (cinahl or (cochrane adj3 trial*) or embase or medline or psyclit or (psycinfo not "psycinfo database") or pubmed or scopus or "sociological abstracts" or "web of science").ab. or ("cochrane database of systematic reviews" or evidence report technology assessment or evidence report technology assessment summary).jn. or Evidence Report: Technology Assessment*.jn. or ((review adj5 (rationale or evidence)).ti,ab. and review.pt.) or meta-analysis as topic/ or Meta-Analysis.pt.

From University of Texas School of Public Health ([Search filters for systematic reviews and meta-analyses](http://libguides.sph.uth.tmc.edu/ovid_medline_filters). Accessed 06 Dec 2013), was used.

### Search strategy, formatted for Psychinfo (OVID)

1806 to August Week 2 2015

| 1 | dementia/ or aids dementia complex/ or alzheimer disease/ or aphasia, primary progressive/ or creutzfeldt-jakob syndrome/ or dementia, vascular/ or diffuse neurofibrillary tangles with calcification/ or frontotemporal lobar degeneration/ or huntington disease/ or kluver-bucy syndrome/ or lewy body disease/ | 55725 |
| --- | --- | --- |
| 2 | exp dementia/ | 57951 |
| 3 | exp Neuropsychological Assessment/ | 14779 |
| 4 | exp Cognitive Assessment/ | 3559 |
| 5 | dementia.ti,ab. | 46844 |
| 6 | alzheimer*.ti,ab. | 44055 |
| 7 | 1 or 2 or 3 or 4 or 5 or 6 | 90002 |
| 8 | systematic*.ti,ab. | 85202 |
| 9 | meta-analysis.ti,ab. | 16369 |
| 10 | "systematic review".ti,ab. | 12154 |
| 11 | (((comprehensive* or integrative or systematic*) adj3 (bibliographic* or review* or literature)) or (meta-analy* or metaanaly* or "research synthesis" or ((information or data) adj3 synthesis) or (data adj2 extract*))).ti,ab,id. or ((review adj5 (rational or evidence)).ti,ab,id. and "Literature Review".md.) or (cinahl or (cochrane adj3 trial*) or embase or medline or psyclit or pubmed or scopus or "sociological abstracts" or "web of science").ab. or ("systematic review" or "meta analysis").md. | 54964 |
| 12 | 8 or 9 or 10 or 11 | 121571 |
| 13 | diagnosis/ | 36489 |
| 14 | brief cognitive tests.ti,ab. | 34 |
| 15 | cognitive screen*.ti,ab. | 708 |
| 16 | ("screening test*" adj2 (dement* or alzheimer*)).ti,ab. | 132 |
| 17 | cog*.ti,ab. | 347512 |
| 18 | 14 or 15 or 16 or 17 | 347557 |
| 19 | 7 and 12 and 13 and 18 | 102 |

N.B. Search filters from University of Texas School of Public Health. [Search filters for systematic reviews and meta-analyses](http://libguides.sph.uth.tmc.edu/ovid_psycinfo_filters). Accessed 06 Dec 2013. [Ovid] incorporated as they added ~30 hits.
